# The Dynamic Environment of Crypto Markets: The Lifespan of New Psychoactive Substances (NPS) and Vendors Selling NPS

**DOI:** 10.3390/brainsci8030046

**Published:** 2018-03-16

**Authors:** Elle Wadsworth, Colin Drummond, Paolo Deluca

**Affiliations:** 1 School of Public Health and Health Systems, University of Waterloo, Waterloo, ON N2L 3G1, Canada; 2 National Addiction Centre, Institute of Psychiatry, Psychology & Neuroscience, King’s College London, London SE5 8BB, UK; colin.drummond@kcl.ac.uk (C.D.); paolo.deluca@kcl.ac.uk (P.D.); 3 South London and Maudsley NHS Foundation Trust, Maudsley Hospital, London SE5 8AZ, UK

**Keywords:** new psychoactive substances, legal highs, darknet, hidden web, crypto market

## Abstract

The Internet has played a major role in the distribution of New Psychoactive Substances (NPS), and crypto markets are increasingly used for the anonymous sale of drugs, including NPS. This study explores the availability of individual NPS and vendors on the crypto markets and considers whether crypto markets are a reliable platform for the sale of NPS. Data was collected from 22 crypto markets that were accessed through the hidden web using the Onion Router (Tor). Data collection took place bimonthly from October 2015 to October 2016 as part of the CASSANDRA (**C**omputer **A**ssisted **S**olutions for **S**tudying the **A**vailability a**N**d **D**ist**R**ibution of novel psycho**A**ctive substances) project. In seven snapshots over 12 months, 808 unique vendors were found selling 256 unique NPS. The total number of individual NPS and vendors increased across the data collection period (increase of 93.6% and 71.6%, respectively). Only 24% (*n* = 61) of the total number of NPS and 4% (*n* = 31) of vendors appeared in every snapshot over the 12 months, whereas 21% (*n* = 54) of NPS and 45% (*n* = 365) of vendors only appeared once throughout the data collection. The individual NPS and vendors did not remain the same over the 12 months. However, the availability of NPS and vendors selling NPS grew. NPS consistently available on crypto markets could indicate popular substances.

## 1. Introduction

New psychoactive substances (NPS) are an emerging phenomenon, and their representation on the hidden web (also known as the darknet) is under-researched. The Internet has played a major role in the distribution of NPS, and drug marketplaces (also known as crypto markets) on the hidden web are increasingly used for the anonymous sale of drugs, including NPS [1,2,3].

The European Union (EU) Early Warning System currently monitors over 650 substances [4]. However, not all substances are a cause of concern, and NPS rise and fall in popularity. Only a few have had sizable prevalence or media attention [5], and research has shown that the legal status of a substance is one of the factors determining why some NPS become popular and others do not [6]. Various legislations have been created to tackle NPS diffusion across Europe and the world. For example, the UK introduced the Psychoactive Substances Act in May 2016, which prohibited the import, export, and supply of psychoactive substances (with some exemptions) but permitted possession [7]. The Act achieved a reduction in UK-based online stores and offline retail stores [8]. As more countries seek to restrict access to NPS, the legal markets could be displaced by alternative routes such as street-level drug dealing or crypto markets [3]. However, movement to the illicit market is dependent on its popularity, and therefore only those NPS with sufficient demand will transition into it [9].

On the hidden web, drugs can be sold and purchased anonymously through crypto markets that use an “eBay”-style system where buyers can publicly review the sellers and therefore build their reputation, attracting future transactions [10,11,12,13,14]. Conversely, negative reviews can discourage buyers and shun unworthy vendors from the crypto markets [11,15]. It is argued that this feedback mechanism and creation of a reputation are vital to the continuing function of the market and also represent an explanation for a vendor’s lifespan [15,16]. To date, the majority of crypto market research has been conducted on the Silk Road [13,17,18,19,20,21], which was closed by law enforcement in 2013 [22]. Christin [20] found that most sellers disappeared before three months, and most items sold for less than three weeks. Christin [20] proposed the theory that short vendor lifespans could be due to lack of stock or to vendors using the “stealth mode”. The stealth mode consists in vendors removing their listings or only selling their listings to a specific customer base [13]. Potentially, the turnover of vendors is a product of the instability of the crypto markets; individual crypto markets are said to be unpredictable and have a frequent turnover, causing instability in the community [23]. Law enforcements have succeeded in closing crypto markets in the past [14,22,24]. However, all successful operations have not stopped the online trade of drugs [25], and the number of vendors has increased [3]. Previous research on crypto markets have shown a small yet definite presence of NPS. Barratt, Ferris, and Winstock [18] examined the use of Silk Road in a sample from the UK, US, and Australia and found that out of the top 20 substances that were purchased, four of these were NPS. In addition, Van Buskirk et al. [3] explored crypto markets in 2015 and concluded that around one-fifth of vendors offered NPS.

Crypto markets are increasingly being used for the sale and purchase of drugs, regardless of the instability both the markets and the vendors show. To date, research has not focussed specifically on the lifespan of vendors selling NPS, substances whose popularity and availability are unpredictable. This paper, therefore, aims to explore the lifespan of individual NPS sold on the crypto markets, explore the lifespan of individual vendors selling NPS on the crypto markets, and consider whether crypto markets are a reliable platform for the sale of NPS.

## 2. Methods

The study was part of the CASSANDRA (**C**omputer **A**ssisted **S**olutions for **S**tudying the **A**vailability a**N**d **D**ist**R**ibution of novel psycho**A**ctive substances) project [26]. The crypto markets on the hidden web were accessed through Tor (torproject.org, 501(c)(3), The Tor Project, Inc., Cambridge, MA, USA), and the data were collected bimonthly over 12 months in October, December (2015), February, April, June, August, and October (2016). The data were collected over two days in each of the seven snapshots. The crypto markets that were included sold NPS, were conducted in English, and had an open registration at the time of collection. The crypto markets present over the 12 months of data collection fluctuated because of crypto markets opening, adding NPS to their sales, or closing following exit scams or law enforcement. A table of the crypto markets included in this study can be found elsewhere [27].

Data were collected on each crypto market for NPS being sold that was visible to the researchers. The data collected were: name of the NPS (not including conventional illicit drugs, steroids, or prescription drugs), name of the vendor selling NPS, and name of the crypto market used by the vendor. Some NPS were sold under various aliases; these were categorised by their most common name. Furthermore, branded NPS that were commonly found on the visible Internet were categorised according to their contents. All analyses were performed using Microsoft Excel 2016 (Microsoft Office 2016, Microsoft, Redmond, WA, United States), unless otherwise specified.

All users on crypto markets were anonymous. The research was purely observational and did not involve interaction with either buyers or sellers. The study was approved by King’s College London PNM Research Ethics reference number: LRS-15/16-3084 as part of the CASSANDRA project.

## 3. Results

**Figure 1:**
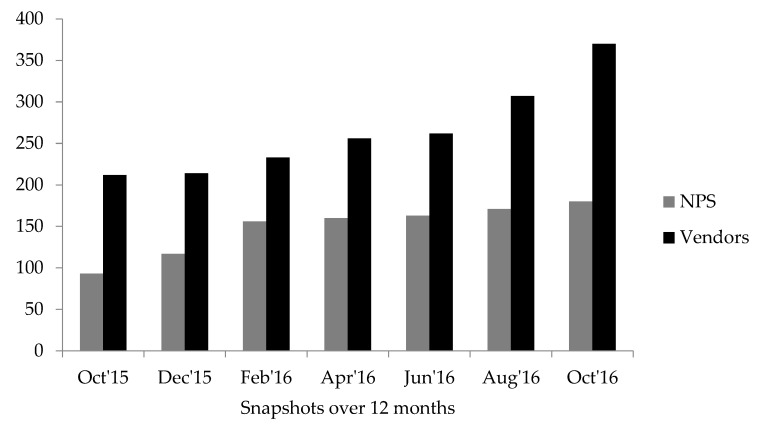
Number of NPS and vendors in each month of data collection (seven snapshots in 12 months)*.* NPS: New Psychoactive Substances.

The total number of individual NPS and vendors increased across the seven snapshots in the data collection period (increase of 93.6% and 71.6%, respectively) (Figure 1). Over 12 months, a total of 808 individual vendors were found selling 256 individual NPS.

**Figure 2:**
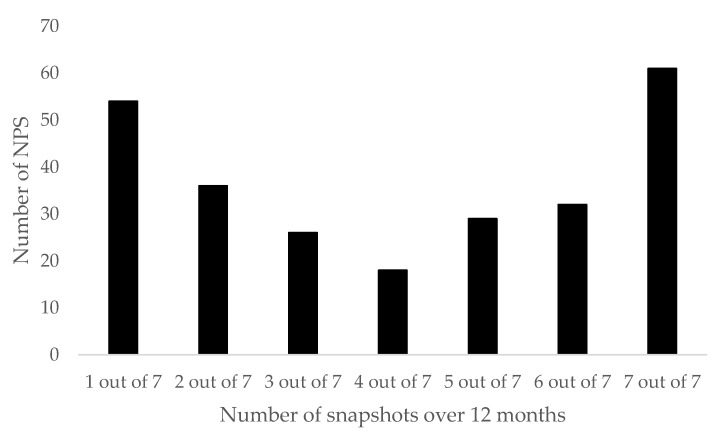
The number of NPS available across the seven snapshots (12 months) of data collection. NPS: New Psychoactive Substances.

A total corresponding to 21% (*n* = 54) of the NPS reported over the 12-month period only appeared in one snapshot (Figure 2). Fourteen percent (*n* = 36) of the total number of NPS appeared in two of the seven snapshots. Ten percent (*n* = 26) appeared in three of the seven snapshots, 7% (*n* = 18) appeared in four of the seven snapshots, 11% (*n* = 29) appeared in five of the seven snapshots, and 13% (*n* = 32) appeared in six of the seven snapshots. In addition, 24% (*n* = 61) of the total number of NPS appeared in each of the seven snapshots over the 12-month period (Table 1).

**Figure 3:**
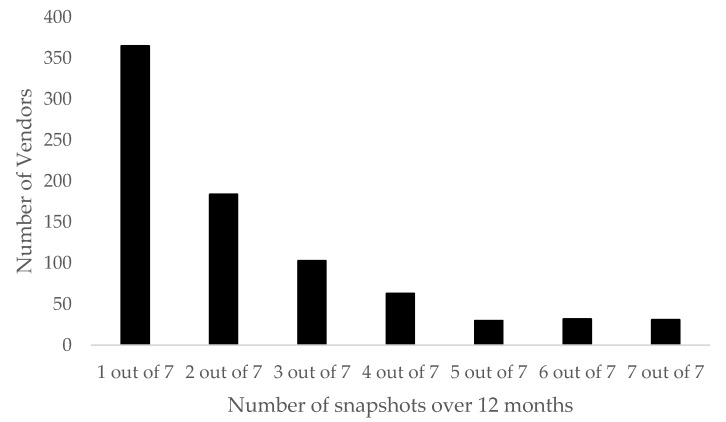
The number of vendors available across the seven snapshots (12 months) of data collection.

Almost half of all vendors (45%, *n* = 365) appeared only once over the 12-month period (Figure 3). Twenty-three percent (*n* = 184) of the total number of vendors appeared in two snapshots over the 12-month period, 13% (*n* = 103) appeared in three snapshots, 8% (*n* = 63) appeared in four snapshots, 4% (*n* = 30) appeared in five snapshots, 4% (*n* = 32) appeared in six snapshots, and only 4% (*n* = 31) of the total number of vendors appeared in all seven snapshots.

## 4. Discussion

The total number of individual NPS and vendors increased over the 12-month duration of the study. Almost half of the total number of vendors had a lifespan of only a few months, and the NPS advertised fluctuated over the study. One-fifth of NPS were available for the entirety of the study, whereas just under one-quarter were only available for a few months before disappearing. From the results of this study, the contents of the NPS market on the crypto markets varied, but a market was consistently available.

Research has shown that the use of crypto markets and the availability of substances including NPS have increased in recent years [3,20,24]. The results from this study complement these findings as the availability of NPS increased over the data collection period. The increased availability of NPS on the crypto markets suggests that NPS are being purchased via this method and that crypto markets are seen as a viable platform for the sale of NPS. This is necessary to observe because of the changing legislations surrounding these substances in countries around the globe, such as the ban in the UK that was implemented during this study [7]. Legal markets will look to migrate to other platforms.

This study found that 256 different NPS were available for purchase over the 12 months of the study, and nearly one-quarter of NPS remained available on the crypto markets, suggesting that these substances could be the most popular NPS. The majority of the NPS that were available in every snapshot were cathinones and phenethylamines (categorised according to [5]). However, there was frequent turnover throughout the study, whereby some NPS were available for purchase for a few months and then disappeared from availability. This pattern may have mirrored changes in popularity within the overall NPS market, fluctuating because of legality, ease of access, or similarities to traditional drugs [4,9]. It is possible that certain NPS were removed from the market because limited stocks were available to the vendors or because the demand of a specific NPS did not match up to its availability. Another possibility is that the feedback from the customers about the NPS was negative, and the stock was removed [15].

Almost half of the vendors were only captured in one snapshot over the course of the data collection, and the number of vendors who appeared in the data reduced as the snapshots increased (Figure 3). These findings show that the lifespan of the vendors was relatively short, and just under half of the vendors were available for under four months, mirroring what was found in the literature [20,28]. There are potential reasons for a short lifespan; research has suggested that vendors could only be selling their limited stock, or that vendors move into stealth mode after they have gathered a sufficient client base [13,20]. Furthermore, the crypto market system is built on trust and reputation; if vendors do not build an adequate reputation, they may remove themselves from the market either indefinitely or only apparently by setting up new accounts [15,28]. Only 4% of vendors remained available in every snapshot over the 12 months of this study. Perhaps, these consistent vendors were highly reputable vendors or vendors with a loyal customer base [28].

This study has several limitations. As this study was exploratory and lacked contact with the vendors, we were unable to verify whether the substances advertised were the same substances being purchased and received by the customers. Because of anonymity, another limitation is that studies like the present one are unable to capture how many vendor profiles are controlled by one person or if the number of vendors that are only available for a few months before disappearing are, in fact, opening and closing various accounts [29].

## 5. Conclusions

Online sales hold a predominant portion of the NPS market, and crypto markets are an emerging source of sale. NPS sold on crypto markets should be monitored to track the popularity and availability of specific substances. Our study found that vendors selling NPS had short lifespans, with half of the vendors only appearing once in the data collection. Individual NPS had longer lifespans, however not all NPS were available over the study period; only a quarter of the total NPS were available in all snapshots over the year. Regardless of the fluctuation of the vendors in the NPS market on the crypto markets, the number of NPS and vendors selling NPS increased over the year. The NPS that consistently appeared across the 12 months of data collection may suggest what substances are popular with the consumers. Crypto markets are therefore to be monitored for the sale of NPS, especially in light of the tightening regulations being applied in various countries.

## Figures and Tables

**Table 1:** New Psychoactive Substances that were available in every snapshot over 12 months of data collection (*n* = 61).

Benzodiazepine Analogue	Cathinone	Dissociative	Opioids	Phenethylamine	Synthetic Cannabinoid	Tryptamine	Other
Etizolam (Etilaam, Etizola, Eticalm)	3-CMC (3-chloromethcathinone)	3-MeO-PCP (3-methoxyphencyclidine)	Fu-F (furanylfentanyl)	25B-NBOMe (N-bombs, cimbi-36)	AB-CHMINACA (AB-C)	4-AcO-DMT (o-acetylpsilocin, psilacetin)	1P-LSD (NP-LAD, 1-Propionyl-d-lysergic acid diethylamide)
	3-MMC (3-methylmethcathinone, 3-mephedrone)	DXE (deschloroketamine, DCK, 2’oxo-PCM)	Kratom (ketum, kratum)	25C-NBOMe (N-bombs, cimbi-82)	MAM-2201 (4′-methyl-AM-2201, 5′′-fluoro-JWH-122)	4-HO-MET (metocin, methylcybin)	AL-LAD (6-allyl-6-nor-LSD)
	4-CMC (4-chloromethcathinone, clephedrone)		W-18 (4-chloro-*N*-[1-[2-(4-nitrophenyl)ethyl]-2piperidinylidene]benzenesulfonamide)	25D-NBOMe (N-bombs)	NM-2201 (CBL-2201)	4-HO-MiPT (miprocin)	LSA (ergine, d-lyseramide, LAA, LA-111)
	4-EMC (4-ethylmethcathinone)			25i-NBOMe (N-bombs, cimbi-5)	“Spice”	5-MeO-DALT (*N,N*-Diallyl-5-methoxytryptamine, Foxtrot)	MPA (methylthienylpropamine, methiopropamine, methedrene, syndrax)
	4-FMC (4-fluoromethcathinone, flephedrone)			2CB (Tripstacy)		5-MeO-MiPT (5-Methoxy-*N*-methyl-*N*-isopropyltryptamine, Moxy)	MXP (methoxphenidine, 2-MeO-diphenidine)
	4F-PV8 (4-Fluoro-α-PHPP, para-fluoro-PV8)			2CE (Tripstacy)			Salvia
	4-MEC (4-methylethcathinone)			2CI (Tripstacy)			
	4-MMC (mephedrone, Mcat, meowmeow, bubble, drone)			2CP (Tripstacy)			
	5-ME (5-methyl-ethylone)			2FA (2-fluoroamphetamine, PAL-353)			
	a-PHP (alpha-PHP, PV7, a-pyrrolidinohexiophenone)			2FMA (2-fluoromethamphetamine)			
	a-PVP (a-pyrrolidinopentiophenone, flakka, k-prolintane, O-2387)			3-FPM (3-fluorophenmetrazine, PAL-593)			
	Dibutylone (bk-DMBDB, booty)			4FA (4-FMP, PAL-303, Flux, PFA)			
	Dimethocaine (DMC, larocaine)			5-EAPB (1-(benzofuran-5-yl)-*N*-ethylpropan-2-amine)			
	Ethylone			5-MAPB (1-(benzofuran-5-yl)-*N*-methylpropan-2-amine)			
	MDPV (NRG-1, methylenedioxypyrovalerone)			6-APB (6-(2-aminopropyl)benzofuran)			
	Methylone (M1, MDMC, bk-MDMA)			DOC (4-Chloro-2,5-dimethoxyamphetamine)			
	PV9 (a-POP, alpha-POP)			DOM (2,5-Dimethoxy-4-methylamphetamine)			
	TH-PVP (3′,4′-tetramethylene-α-Pyrrolidinovalerophenone)			ephedrine			
				Ethylphenidate (EPH)			
				MAL (methallylescaline)			
				MDA (3,4-Methylenedioxyamphetamine)			
